# Spatiotemporal signal space separation for regions of interest: Application for extracting neuromagnetic responses evoked by deep brain stimulation

**DOI:** 10.1002/hbm.26602

**Published:** 2024-01-30

**Authors:** Ashwini Oswal, Bahman Abdi‐Sargezeh, Abhinav Sharma, Tolga Esat Özkurt, Samu Taulu, Nagaraja Sarangmat, Alexander L. Green, Vladimir Litvak

**Affiliations:** ^1^ MRC Brain Network Dynamics Unit University of Oxford Oxford UK; ^2^ Nuffield Department of Clinical Neurosciences University of Oxford Oxford UK; ^3^ The Wellcome Centre for Human Neuroimaging University College London London UK; ^4^ Department of Neurology John Radcliffe Hospital Oxford UK; ^5^ Graduate School of Informatics Middle East Technical University Ankara Turkey; ^6^ Department of Physics University of Washington Seattle Washington USA; ^7^ Institute for Learning and Brain Sciences University of Washington Seattle Washington USA

**Keywords:** magnetoencephalography, source leakage correction, spatiotempotal signal separation

## Abstract

Magnetoencephalography (MEG) recordings are often contaminated by interference that can exceed the amplitude of physiological brain activity by several orders of magnitude. Furthermore, the activity of interference sources may spatially extend (known as source leakage) into the activity of brain signals of interest, resulting in source estimation inaccuracies. This problem is particularly apparent when using MEG to interrogate the effects of brain stimulation on large‐scale cortical networks. In this technical report, we develop a novel denoising approach for suppressing the leakage of interference source activity into the activity representing a brain region of interest. This approach leverages spatial and temporal domain projectors for signal arising from prespecified anatomical regions of interest. We apply this denoising approach to reconstruct simulated evoked response topographies to deep brain stimulation (DBS) in a phantom recording. We highlight the advantages of our approach compared to the benchmark—spatiotemporal signal space separation—and show that it can more accurately reveal brain stimulation‐evoked response topographies. Finally, we apply our method to MEG recordings from a single patient with Parkinson's disease, to reveal early cortical‐evoked responses to DBS of the subthalamic nucleus.

## Graphical abstract

Here, we develop methodological approaches for both validating and improving the estimation accuracy of deep brain stimulation‐evoked responses using magnetoencephalography (MEG). By leveraging spatial and temporal projectors, our proposed method can also allow for the alleviation of ‘source leakage’, which is major limitation of MEG source estimation.

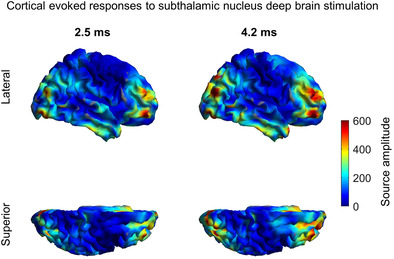

## 1 INTRODUCTION

Magnetoencephalography (MEG) is a powerful technique for interrogating brain network dynamics (Baillet et al., 2001; Brookes et al., 2011; De Pasquale et al., 2010; Hämäläinen et al., 1993; Hipp et al., 2012). Disturbances of network dynamics are increasingly recognised to contribute to the pathophysiology of neurological conditions such as Parkinson's disease (PD) (Oswal et al., 2013). MEG has also been used to investigate how therapeutic interventions including brain stimulation techniques can modulate large‐scale brain networks (Abbasi et al., 2018; Airaksinen et al., 2011; Hartmann et al., 2018; Litvak et al., 2010; Litvak et al., 2011). Deep brain stimulation (DBS) is one example of such a technique that is used to treat PD, which involves electrically stimulating basal ganglia structures such as the subthalamic nucleus (STN) (Limousin & Foltynie, 2019; Lozano et al., 2019).

A significant challenge of MEG is its vulnerability to contamination from physiological and non‐physiological artefacts. MEG recordings during brain stimulation are particularly challenging since the magnitude of the stimulation signal far exceeds that of brain activity of interest (Oswal, Beudel, et al., 2016; Oswal, Jha, et al., 2016). A related problem is that source estimation techniques are ill‐posed, meaning that a few hundred channels cannot sufficiently discriminate brain activity in many thousands of voxels. This issue can partly contribute to a problem known as ‘source leakage’, whereby reconstructions of true dipolar point sources are spatially spread over several sometimes spatially segregated voxels, thereby leading to artefactual correlations between inferred brain sources (Brookes et al., 2012; Colclough et al., 2015; Hauk et al., 2022). Importantly for brain stimulation studies using MEG, the leakage of stimulation artefacts can preclude the accurate estimation of the effects of stimulation on a brain region of interest (ROI).

Spatiotemporal signal space separation (tSSS) (Taulu et al., 2004; Taulu & Kajola, 2005; Taulu & Simola, 2006) is a useful approach for suppressing interference sources that are external to the MEG sensor array. In tSSS, a spatial filter derived from spherical harmonic functions is first used to decompose the data into components corresponding to source locations inside and outside the sensor array. A temporal projector is subsequently applied to remove signal components that are highly correlated—representing artefacts—between these two subspaces. This method has an important shortcoming in that it does not explicitly account for the fact that artefactual sources—whose activities can leak into the reconstructed activity of brain ROIs—can originate from within the brain. Here, we overcome this limitation by developing an extension of the tSSS approach for spherical or cuboidal anatomical regions of interest. A spatial filter derived from spherical harmonics is first used to divide the data into components originating from within and from outside an ROI (Ozkurt et al., 2006; Özkurt et al., 2009). Temporal projection is subsequently applied to remove instantaneously correlated (at zero‐lag) components representing leakage between these two subspaces. Interestingly, our approach bears resemblance to leadfield‐based spatial filtering (beamspace) algorithms (Cai et al., 2019; Oswal et al., 2014; Rodríguez‐Rivera et al., 2006; Sekihara et al., 2016; Sekihara et al., 2018), but a key difference is that it is independent of the computation of a forward model.

A number of different algorithms have been developed and tested for their ability to suppress brain stimulation artefacts during MEG recordings (Abbasi et al., 2016; Kandemir et al., 2020; Oswal et al., 2014; Oswal, Jha, et al., 2016). Of these, only tSSS has been successfully employed for the estimation of brain stimulation‐evoked responses, which are short‐lived and tightly correlated to high‐amplitude stimulation pulses and subsequent artefacts (Bahners et al., 2023; Hartmann et al., 2018; Spooner et al., 2023). Here, we build on this work and compare the utility and accuracy of tSSS and our new approach, ROI‐based tSSS (ROI‐tSSS), for estimating DBS‐evoked responses using phantom and patient recordings.

## 2 METHODS

### 2.1 Phantom recording for validating estimation accuracy of stimulation‐evoked responses

We have previously described a phantom experiment to characterise DBS artefacts during MEG recordings (Oswal, Jha, et al., 2016). We used a CTF current dipole phantom, comprising a spherical plastic container holding saline (with an inner diameter of 13 cm), in which a dipolar source was immersed. The dipolar source was driven by a 27 Hz sinusoidal signal, to mimic activity within the physiological beta range, and its amplitude was set to 6.7 μA to create a peak in the power spectrum that slightly exceeded the background noise level. To allow for monopolar stimulation, a DBS electrode (Medtronic model 3389, with four platinum‐iridium contacts with centre‐to‐centre separation of 2 mm) and an anodal reference electrode were additionally implanted into the phantom. Monopolar DBS was administered using a Medtronic external stimulator (type 3628) between contact 1 (cathode) of the DBS electrode and the anodal reference. DBS was delivered at frequencies of 0 (no stimulation condition), 5, 20 or 130 Hz (pulse width = 60 μs; fixed voltage, amplitude = 3 V). We simulated evoked responses using this data by epoching continuous data segments into trials that were locked to a specific phase of the 27 Hz source.

To create a more realistic phantom simulation, two additional and related artefacts were incorporated to replicate what is typically observed in patient recordings. First, low‐amplitude movements were simulated to replicate arterial pulsations and slight head movements resulting from each heartbeat. These movements were generated by placing an inflatable balloon under the phantom, which was periodically inflated with air 60 times per minute using custom‐made electronics. We ensured that the resulting movements of the phantom were similar in terms of their magnitude to head movements that are observed in patient recordings. To simulate the second type of artefact, two ferromagnetic extension wires identical to those used in patient recordings were positioned on the spherical surface of the phantom. We have previously shown that movements of the ferromagnetic wires are related to arterial pulsations and that the interaction of these two phenomena is a major source of artefacts (Litvak et al., 2010; Oswal, Jha, et al., 2016).

MEG recordings—sampled at 2.4 kHz using a CTF 275 channel system—were first denoised using either tSSS or our novel method, ROI‐tSSS (see below). A high pass filter (5 Hz) was subsequently applied before epoching. Trial data were averaged, and topographies of the simulated evoked response were compared for the different combinations of stimulation conditions and denoising approaches. A control condition (‘Standard’) where no denoising was applied is also included. Simulated dipole time courses were reconstructed for visualisation using the *ft_dipolefitting* function in FieldTrip (Oostenveld et al., 2011).

### 2.2 Extending spatiotemporal signal separation for regions of interest

tSSS uses a spatial filter to decompose an N channel MEG signal, b into two components ($b_{\text{in}}$ and $b_{\text{out}}$) corresponding to source locations inside and outside the MEG sensor array (Taulu & Simola, 2006).

(1) $$b=\sum_{l=1}^{L_{\text{in}}}\sum_{m=-l}^{l}\alpha_{\mathrm{lm}}a_{\mathrm{lm}}+\sum_{l=1}^{L_{\text{out}}}\sum_{m=-l}^{l}\beta_{\mathrm{lm}}b_{\mathrm{lm}}=b_{\text{in}}+b_{\text{out}}$$

In Equation (1), $a_{\mathrm{lm}}$ and $b_{\mathrm{lm}}$ are SSS basis functions, which depend on sensor geometry and are derived from the gradients of spherical harmonic functions in spherical coordinates. $L_{\text{in}}$ and $L_{\text{out}}$ govern dimensions of the SSS basis set and are prespecified as 8 and 6, respectively, (Taulu & Simola, 2006). Equation (1) expressed in matrix form is as follows:

(2) $$b=\mathrm{Sx}=\left[S_{\text{in}}S_{\text{out}}\right]\left[\begin{array}{c} x_{\text{in}}\\ x_{\text{out}} \end{array}\right]$$

$$S_{\text{in}}=\left[a_{1,-1}\ldots a_{L_{\text{in},}L_{\text{in}}}\right]$$

$$S_{\text{out}}=\left[b_{1,-1}\ldots b_{L_{\text{out},}L_{\text{out}}}\right]$$

$$\;x_{\text{in}}={\left[\alpha_{1,-1}\ldots \alpha_{L_{\text{in},}L_{\text{in}}}\right]}^{T}$$

$$x_{\text{out}}={\left[\beta_{1,-1}\ldots \beta_{L_{\text{out},}L_{\text{out}}}\right]}^{T}$$

Equation (2) reveals that SSS coefficients, $x_{\text{in}}$ and$\;x_{\text{out}}$ can be computed from the pseudoinverse of the SSS basis ($S^{*}$) set and the data as follows:

(3) $$\hat{x}=\left[\begin{array}{c} {\hat{x}}_{\text{in}}\\ {\hat{x}}_{\text{out}} \end{array}\right]=S^{*}b$$

Estimates of $x_{\text{in}}$ and$\;x_{\text{out}}$ can then be used to estimate $b_{\text{in}}$ and $b_{\text{out}}$:

(4) $${\hat{b}}_{\text{in}}=S_{\text{in}}{\hat{x}}_{\text{in}}$$

$${\hat{b}}_{\text{out}}=S_{\text{out}}{\hat{x}}_{\text{out}}$$

Building on previous results (Ozkurt et al., 2006; Özkurt et al., 2009), we show that manipulating the SSS coefficient $x_{\text{in}}$ can filter the SSS signal into separate components originating from within and from outside a spherical brain ROI (e.g., motor cortex; see Supporting Information S1 for further details). The modified SSS coefficient for the ROI, ${\hat{x}}_{\mathrm{ROI}}$, is given by:

(5) $${\hat{x}}_{\mathrm{ROI}}=G_{\mathrm{ROI}}{\hat{x}}_{\text{in}}$$

where

(6) $${\hat{x}}_{\mathrm{ROI}}={\left[{\alpha^{\mathrm{ROI}}}_{1,-1}\ldots {\alpha^{\mathrm{ROI}}}_{L_{\text{in},}L_{\text{in}}}\right]}^{T}$$

(7) $$G_{\mathrm{ROI}}=\mathrm{diag}\left(\left(\frac{\left(R^{5}-r^{5}\right)r^{2l-2}}{R^{2l+3}-r^{2l+3}}\right)\frac{2l+3}{5}\right).$$

In Equation (7), $G_{\mathrm{ROI}}$ is a diagonal matrix with dimensions ${\left(L_{\text{in}}+1\right)}^{2}-1$ by ${\left(L_{\text{in}}+1\right)}^{2}-1$. This dimensionality arises due to the double summation in Equation (1), which results in a number of terms for ${\hat{x}}_{\text{in}}$ that is equal to the sum of the series of odd numbers from 3 to $2L_{\text{in}}+1$. It can be shown that, l for each diagonal element $G_{\mathrm{ROI}}\left(n,n\right)$ is given by the floor function of the square root of n, $\left\lfloor \sqrt{n}\right\rfloor$ (Özkurt et al., 2009).

**FIGURE 1:**
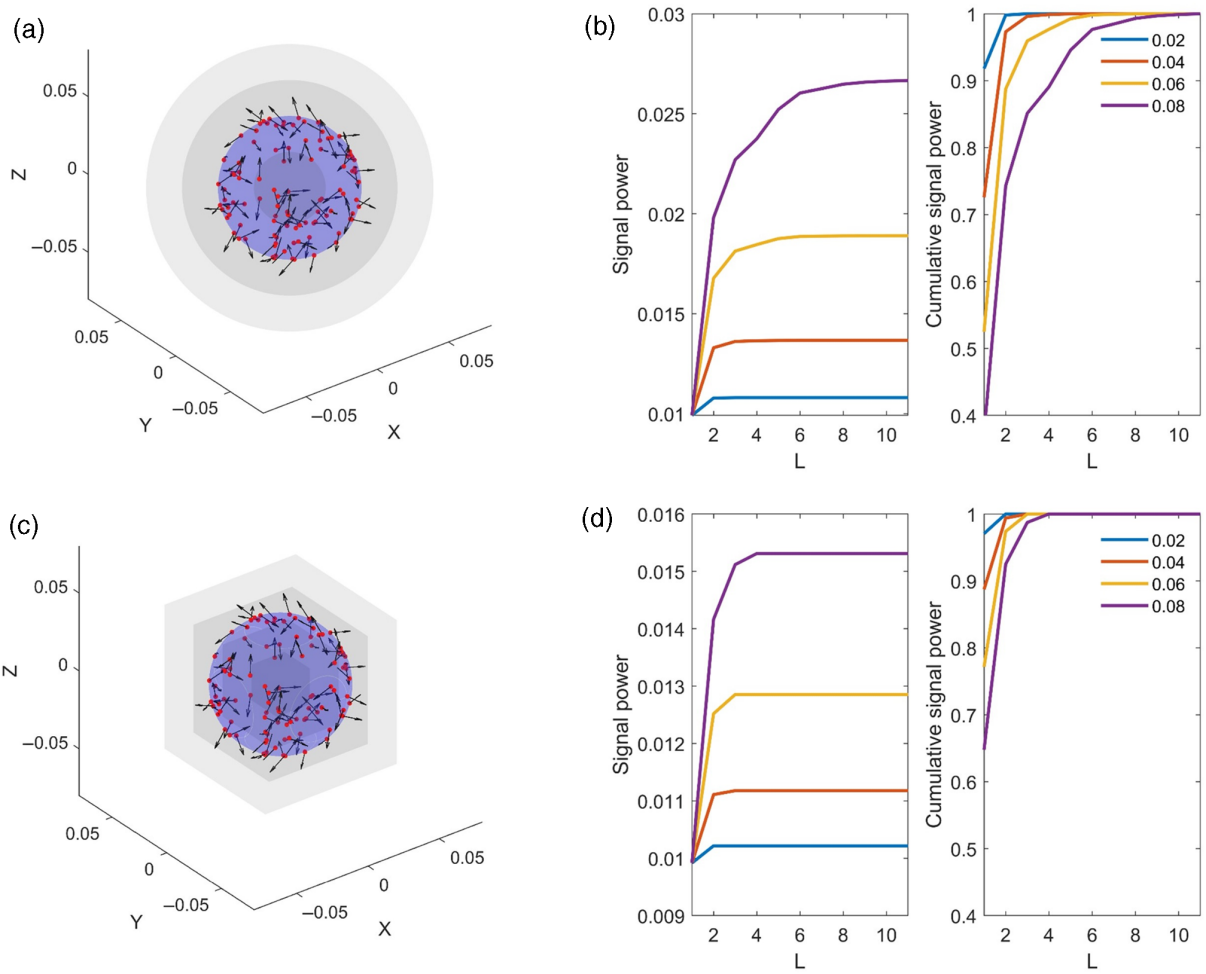
The effect of ROI size on signal recovery for spherical and cubic ROIs. Upper panel: (a) highlights the setup of the simulation with 100 randomly oriented sources on the surface of the blue sphere at a distance of 0.04 m from the expansion origin. The sensor distance from the origin, *R* is fixed at 0.08 m. Grey spheres represent spherical boundaries of the ROIs that were tested (b) the effect of ROI radius on signal power and cumulative signal power. When the ROI does not encompass the simulated sources a small proportion of the signal power is recovered and there is little dependence on L. Signal power recovery and the dependence on L increase as the ROI radius approaches *R*. Lower panel (c and d) is as per upper panel, except for a cubic rather than a spherical ROI. For cubic ROIs, we selected the dimensions of the side such that the body diagonal was equal to the diameter of the corresponding sphere in (a).

Importantly in Equation (7), r represents the radius of the ROI and R is the radius of the sensor array from the SSS expansion origin, added to the distance between the SSS expansion origin and the centre of the ROI (Özkurt et al., 2009) (see Figure 1a).

Similarly, the modified SSS coefficient for regions outside the ROI, ${\hat{x}}_{\neg \mathrm{ROI}}$, is as follows:

(8) $${\hat{x}}_{\neg \mathrm{ROI}}=G_{\neg \mathrm{ROI}}{\hat{x}}_{\text{in}}$$

Here, the logical negation symbol, ¬ is used in the subscript to indicate regions outside of the ROI. $G_{\neg \mathrm{ROI}}$ is a diagonal matrix with the same dimensions as $G_{\mathrm{ROI}}$ and is given by:

(9) $$G_{\neg \mathrm{ROI}}=\mathrm{diag}\left(\left(\frac{r^{2L_{\text{in}}-2l}R^{2l+3}-r^{2L_{\text{in}}+3}}{R^{2L_{\text{in}}+3}-r^{2L_{\text{in}+}3}}\right)\frac{2L_{\text{in}}+3}{2l+3}\right)$$

Consequently, estimates of the signal originating from the volume of the ROI (${\hat{b}}_{\mathrm{ROI}}$) and from the brain volume external to it (${\hat{b}}_{\neg \mathrm{ROI}}$) are given by:

(10) $${\hat{b}}_{\mathrm{ROI}}=S_{\text{in}}{\hat{x}}_{\mathrm{ROI}}$$

(11) $${\hat{b}}_{\neg \mathrm{ROI}}=S_{\text{in}}{\hat{x}}_{\neg \mathrm{ROI}}$$

Following the spatial filtering step in tSSS, a temporal filter is applied to remove correlated signal components (representing artefacts) in both ${\hat{b}}_{\text{in}}$ and ${\hat{b}}_{\text{out}}$ (Taulu & Simola, 2006). In ROI‐tSSS, we apply temporal filtering to remove from ${\hat{b}}_{\mathrm{ROI}}$ components that are correlated between ${\hat{b}}_{\mathrm{ROI}}$ and ${\hat{b}}_{\neg \mathrm{ROI}}$ (see Supporting Information S1). This procedure removes zero‐lag correlated signal components from brain ROI activity and, therefore, offers source leakage correction.

The above procedure assumes spherical ROIs. To achieve improved control of brain volumes encompassed by an ROI, we extended our algorithm to include cuboidal ROIs (see Methods in Supporting Information S1).

### 2.3 Construction of power functions

It has previously been shown (Taulu & Kajola, 2005) that the dependence of signal power recovery on l is given by:

(12) $$\sqrt{\sum_{m}f_{l,m}^{2}\left(r_{\alpha },R,J_{\text{in}}\right)}$$

where m=−l…l and

(13) $$f_{l,m}\left(r_{\alpha },R,J_{\text{in}}\right)=\sqrt{\frac{l}{2l+1}\;}{∭}_{0}^{R}{\left(\frac{r_{\alpha }}{R}\right)}^{l+2}{\mathrm{iX}}_{l,m}^{*}\left(\theta ,\varphi \right).J_{\text{in}}\left(r_{\alpha }\right)\;\mathrm{dr}\;\mathrm{d\theta}\;\mathrm{d\varphi}$$

In Equation (13), R represents the radial distance of the sensor array from the expansion origin, whilst $r_{\alpha }$ is the radius of the source volume. This expansion can be used to determine the optimal value for $L_{\text{in}}$ (see fig. 1 in Taulu & Simola, 2006). We modify this function for regions of interest by scaling by $G_{\mathrm{ROI}}\left(r\right)$, which is a function of the ROI radius, r.

(14) $$f_{l,m}^{\mathrm{ROI}}\left(r,r_{\alpha },R,J_{\text{in}}\right)=G_{\mathrm{ROI}}\left(r\right)\sqrt{\frac{l}{2l+1}\;}{∭}_{0}^{R}{\left(\frac{r_{\alpha }}{R}\right)}^{l+2}{\mathrm{iX}}_{l,m}^{*}\left(\theta ,\varphi \right).J_{\text{in}}\left(r_{\alpha }\right)\;\mathrm{dr}\;\mathrm{d\theta}\;\mathrm{d\varphi}$$

This modified power function allows us to consider the relationship between signal power recovery, r and l. We simulated 100 randomly oriented current dipoles on the surface of a 4 cm ($r_{\alpha }$) sphere, whilst R was fixed to 8 cm. The function $\sqrt{\sum_{m}{f^{2}}_{l,m}^{\mathrm{ROI}}\left(r,r_{\alpha },R,J_{\text{in}}\right)}$ was then computed for different values of r and l. The results of this simulation are shown in Figure 1 for spherical and cubic ROIs (for cubic ROIs we selected dimensions such that the cube fitted perfectly within a sphere of a particular radius). When the ROI does not encompass simulated sources, little signal power is recovered as expected and there is little dependence on l. Signal power recovery and dependence on l increases as r→R. To preserve the mapping between sensor space and source space representations when performing source analysis, we applied the spatial component of the ROI‐tSSS filter to the leadfields using the *spm_eeg_montage* function.

### 2.4 Application of ROI‐tSSS for detecting DBS‐evoked responses

We applied the ROI‐tSSS algorithm to extract cortical‐evoked responses to 5 Hz monopolar STN DBS in a patient with PD (see Methods in Supporting Information S1). LCMV beamformer (van Veen et al., 1997) source localisation was performed for each 5 mm spaced grid point on the 3D brain volume using the SPM DAiSS toolbox (https://github.com/SPM/DAiSS). Data used for beamforming each grid point were processed using the ROI‐tSSS algorithm (spherical ROI with 5 cm radius), before being high pass filtered (5 Hz) and epoched to the onset of DBS pulses. For comparison, we also performed source localisation after processing with tSSS.

For both phantom and patient recordings, three head position indicator (HPI) coils were used to facilitate co‐registration for source reconstruction. We used continuous headtracking and employed a previously described procedure to ensure that estimates of head position were robust to interferences caused by the presence of ferromagnetic wires and DBS pulses (Oswal, Beudel, et al., 2016; Oswal, Jha, et al., 2016). The full details are provided in the aforementioned studies, but the overall idea is based on the fact that if head position is accurately tracked, the pairwise distances between the HPI coils should stay constant within a recording run. Time points where head tracking was lost within a recording run could then be identified and corrected by interpolation, such that the average of the resulting interpolated values would yield a robust estimate of head position. Head and sensor locations were always visually inspected for each subject and compared across runs to make sure that there were no gross outliers or misregistrations.

## 3 RESULTS

### 3.1 Phantom recordings: Improved estimation of evoked response topographies

**FIGURE 2:**
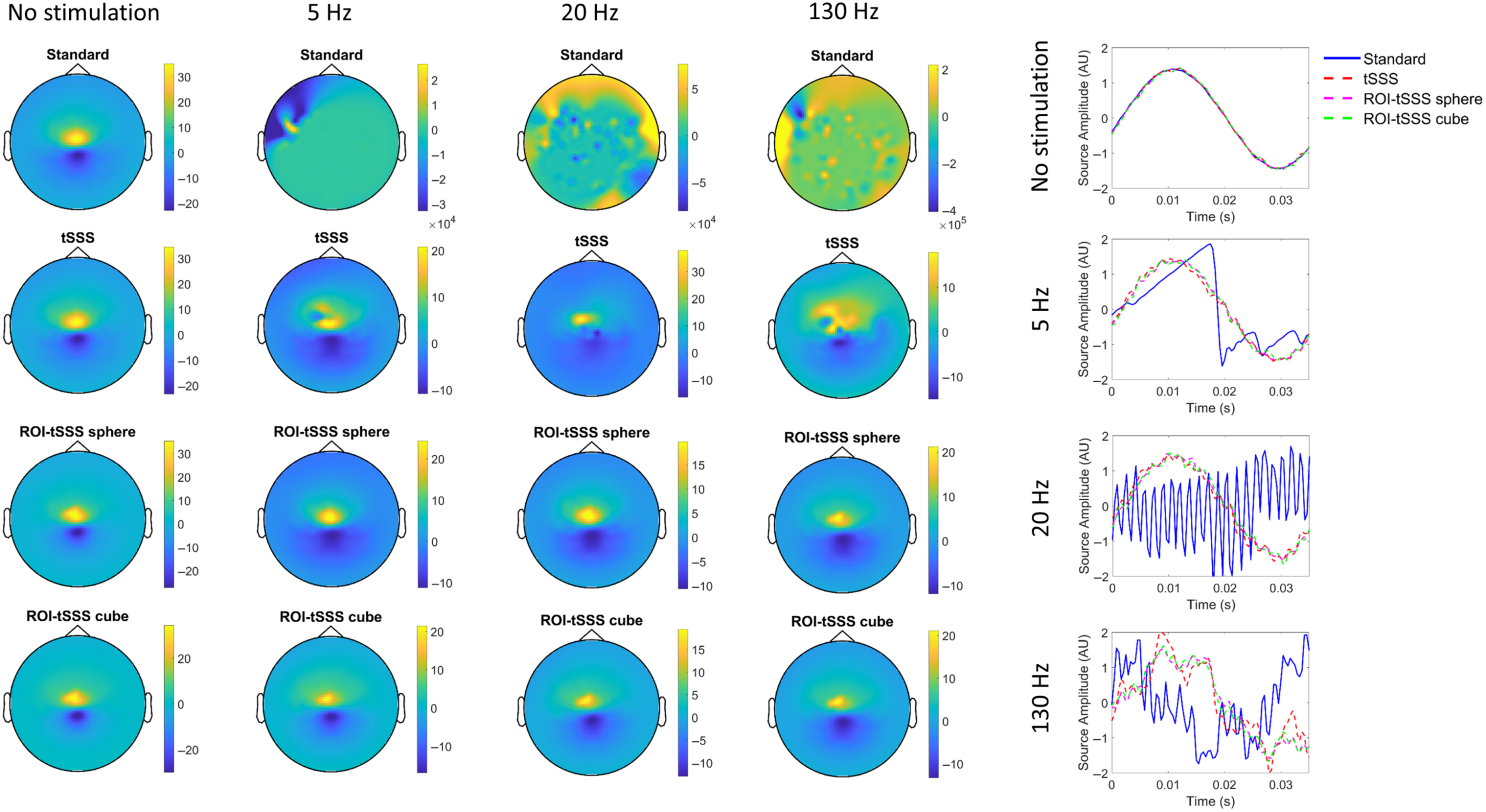
Application of spatiotemporal signal separation approaches to reconstructing evoked responses in a phantom recording. Left: Topographies of the simulated dipole at the four different DBS settings (no stimulation, 5 Hz monopolar DBS, 20 Hz monopolar DBS and 130 Hz monopolar DBS), after pre‐processing the data with one of four different approaches (standard pre‐processing, tSSS, ROI‐tSSS sphere and ROI‐tSSS cube) are shown. The colour bars represent field strength measured in femtoteslas (fT). Plots to the right show the reconstructed time courses of the simulated sinusoid, for each DBS setting and each pre‐processing approach. The ROI‐tSSS sphere and ROI‐tSSS cube approaches reproduce the dipole topography well across all stimulation conditions.

Figure 2 shows the topography of the simulated evoked response for the different monopolar DBS frequencies and pre‐processing approaches. When no denoising is applied (‘Standard’) the topographies are inaccurately reconstructed across all DBS settings. This reflects the fact that monopolar DBS has non‐linear effects that result in artefacts across a range of frequencies (Oswal, Jha, et al., 2016). The ROI‐tSSS approach—using either a spherical or cubic ROI centred on the simulated dipole—provides accurate reconstruction of topographies across all stimulation frequencies.

**FIGURE 3:**
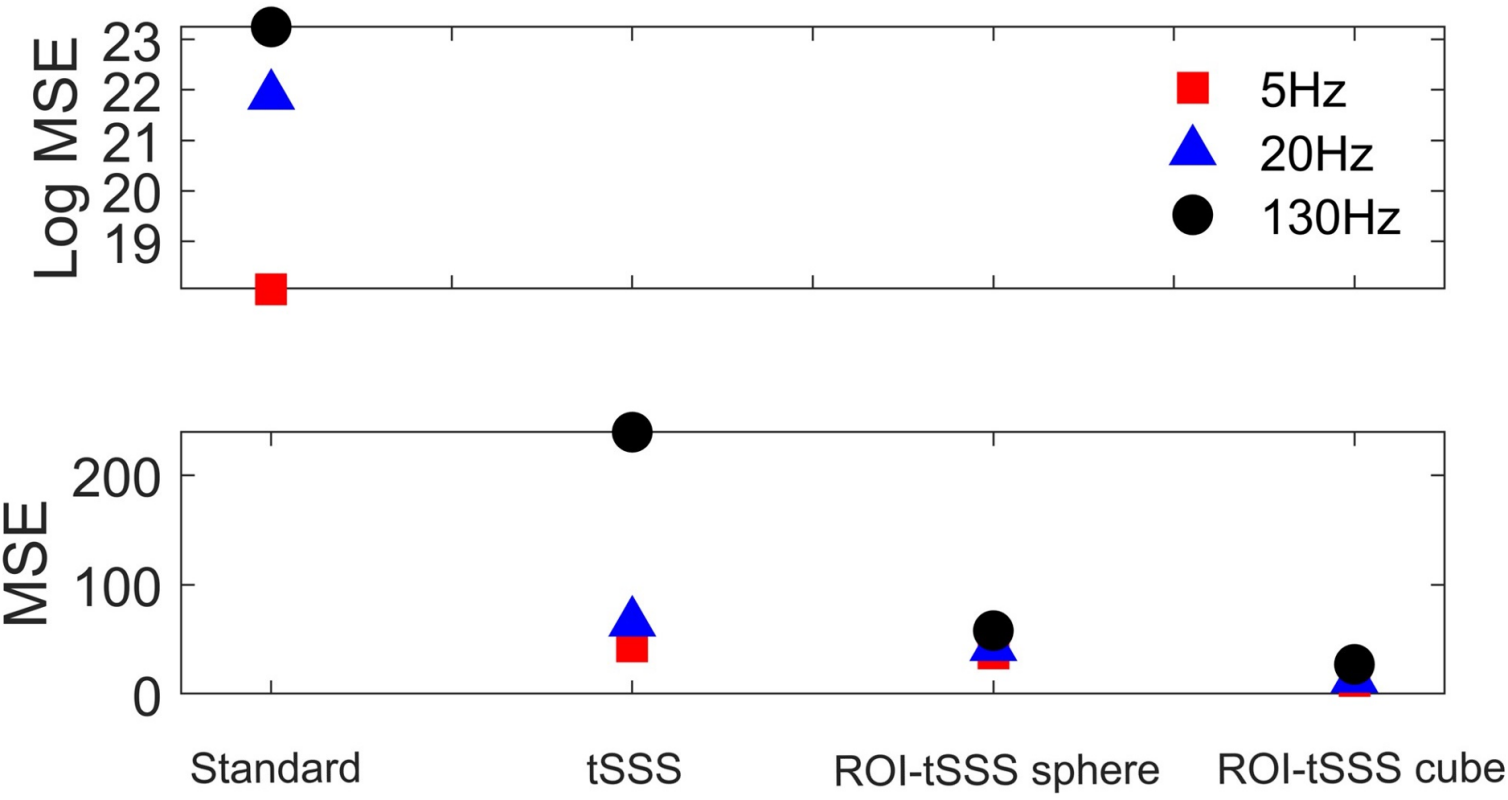
The mean squared error (MSE) of the simulated dipole topography, computed between different DBS conditions and the no stimulation condition is plotted for all four pre‐processing approaches in the phantom recording. The lowest MSE across all stimulation conditions is achieved by the ROI‐tSSS sphere and the ROI‐tSSS cube approaches. The ROI‐tSSS approaches offer the greatest benefit at clinically deployed DBS frequencies of 130 Hz.

For each pre‐processing approach, we computed the mean squared error (MSE) between the sensor timeseries of each stimulation condition and the corresponding no‐stimulation condition (Figure 3). ROI‐tSSS outperforms tSSS for all stimulation conditions with the difference being particularly pronounced at 130 Hz, which is the most commonly employed clinical DBS frequency. Figure 2 also shows estimated time courses for the simulated dipole for the different DBS conditions and pre‐processing approaches.

### 3.2 Patient recording: Effects of STN DBS on cortical networks

**FIGURE 4:**
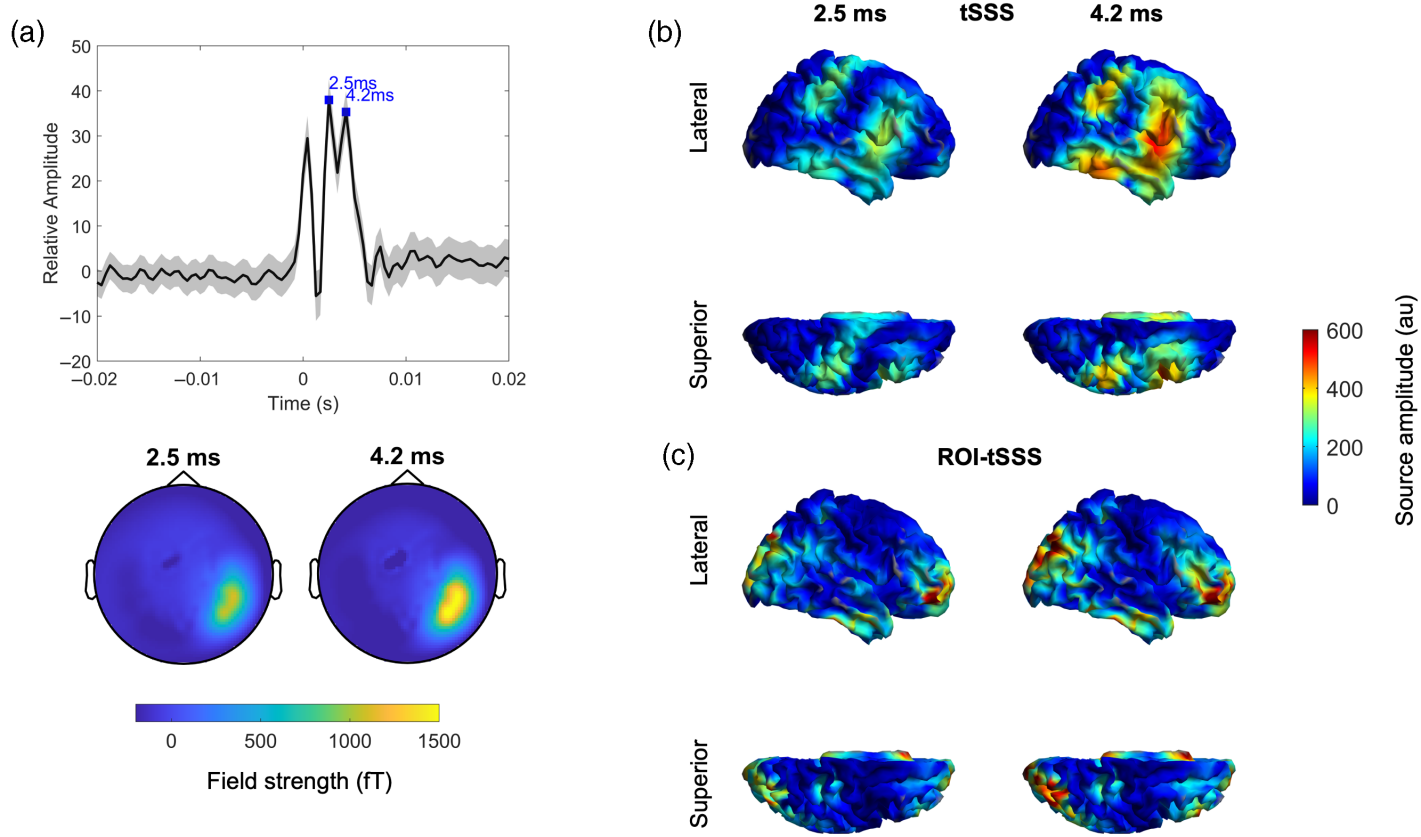
(a) The ROI‐tSSS approach is applied to detect the evoked response to monopolar 5 Hz DBS of the right subthalamic nucleus in a patient with Parkinson's disease. The upper panel shows the sensor level evoked response (averaged across trials and channels after baseline correction relative to a 20 ms window [−0.05 to −0.03 s] prior to the onset of the stimulation pulse at time 0) after constructing a 5 cm region of interest centred on the right motor cortex at MNI co‐ordinates 37–18 53; the lower panel shows the corresponding topography of the evoked response peaks at 2.5 and 4.2 ms. (b and c) Right hemispheric source level evoked response amplitudes at the times of the two peaks are extracted using LCMV beamforming and projected onto a cortical mesh after applying tSSS in (c) and ROI‐tSSS in (d). In (d), for each grid point, the ROI‐tSSS sphere algorithm (with a 5 cm radius) was applied prior to beamforming. There are focal frontal, temporal and parieto‐occipital regions demonstrating high‐amplitude evoked responses.

Figure 4a shows the mean evoked response, averaged over trials and channels, after constructing a spherical ROI around the right motor cortex at MNI coordinates 37 −18 53 (Litvak et al., 2012). There are early peaks after 2.5 and 4.2 ms. The sensor topographies of these two peaks are also shown, revealing the activation of parietal sensors. Finally, LCMV beamformer reconstructed source amplitudes at the timings of the evoked response peaks are displayed on a cortical mesh after processing with tSSS (Figure 4b) and ROI‐tSSS (Figure 4c). Frontal and temporal activations at 2.5 and 4.2 ms are observed with both tSSS and ROI‐tSSS. Interestingly, with ROI‐tSSS, the frontal activations are more anterior—including regions such as the middle and inferior frontal gyri—and there are also activations in parieto‐occipital regions that are not seen with tSSS (see the Discussion section).

## 4 DISCUSSION

The overall goal of this report is to build on and to improve existing methodologies for using MEG to characterise the effects of brain stimulation techniques on large‐scale brain networks in health and disease. To this end, we first developed an extension of the widely used tSSS algorithm for anatomical regions of interest (ROI‐tSSS). ROI‐tSSS involves the construction of separate spatial projectors for a brain ROI and for brain areas outside the ROI. A temporal projector is then used to remove signal components—representing leakage artefacts—that are common to both the ROI and areas outside the ROI. Our approach allows for the specification of both spherical and cuboidal ROIs and may be useful as a leakage suppression approach prior to source reconstruction. In contrast, previous studies addressing the issue of source leakage have proposed post‐hoc time series orthogonalization approaches that are employed following source reconstruction (Brookes et al., 2012; Colclough et al., 2015). Of interest, although ROI‐tSSS is independent of forward model specification, there are related ROI‐based spatial filtering techniques, which rely on eigendecomposition of source leadfields within an ROI (Oswal et al., 2014; Rodríguez‐Rivera et al., 2006; Sekihara et al., 2018). It would be interesting to compare such leadfield‐based approaches to ROI‐tSSS in future studies.

Using phantom recordings, we have shown that ROI‐tSSS performs favourably compared with tSSS for reconstructing a simulated evoked response under different monopolar DBS conditions. It is important, however, to bear in mind certain limitations when considering the results of our phantom recordings.

First, we simulated artefacts that are typically seen in externalised patient recordings. In such cases, the DBS electrodes are temporarily connected to ferromagnetic wires that leave the scalp to allow for research recordings for a few days prior to a second procedure in which the wires are removed and the electrode is connected to a stimulator implanted in the chest wall. While it is possible that the magnetic fields generated during externalised recordings may be different to those generated with the stimulator implanted within the chest wall (see Oswal, Jha, et al., 2016 for a further discussion of this), it is worth pointing out that tSSS has proven to be an effective method for artefact suppression even with implanted devices in situ (Bahners et al., 2023; Spooner et al., 2023). Second, the phantom recordings were designed to reflect a scenario where we could be sure of the ground truth for comparisons between tSSS and ROI‐tSSS. Patient recordings are undoubtedly more complex due to additional physiological artefacts and the fact that the evoked responses to each stimulation pulse will contain multiple interacting sources, whose activity patterns are often correlated (see below). Finally, although in our phantom recordings the simulated evoked response was not temporally locked to the onset of each stimulation pulse, we did include a 130 Hz stimulation frequency, which we have previously shown to result in continuous ringing artefacts between each 7.7 ms spaced stimulation pulse (Oswal, Jha, et al., 2016). This ensured that each simulated evoked response occurred on the background of continuous artefacts and was within 7.7 ms of a stimulation pulse. Interestingly, this time window of 7.7 ms is comparable to the latency of short DBS‐evoked responses (Miocinovic et al., 2018). In this 130 Hz stimulation condition, we observed that ROI‐tSSS provided the greatest improvement in the MSE of topography reconstruction compared with tSSS.

In MEG recordings from a single PD patient undergoing STN DBS, we compared the spatial patterns of evoked responses at the source level after data processing with tSSS and ROI‐tSSS. In both cases, there were early frontal and temporal activations, consistent with previous findings from invasive electrocorticography studies (Chen et al., 2020; Jorge et al., 2022). There were, however, also important differences observed in the spatial activation patterns seen with tSSS and ROI‐tSSS. Frontal activations were more anterior and focal with ROI‐tSSS and there were also parieto‐occipital activations, which could be consistent with the existence of functional connectivity networks between these cortical areas and the STN (Litvak et al., 2011). In addition, sensorimotor cortical activations observed with ROI‐tSSS were significantly smaller and much more focal. These differences may reflect improved leakage mitigation and signal‐to‐noise ratio offered by ROI‐tSSS, which in turn could lead to improved spatial resolution and source estimation.

## CONFLICT OF INTEREST STATEMENT

AO, BA, TEO, ST and VL have no competing interests. NS has received honoraria from Medtronic and support for attending scientific meetings from Medtronic and Boston Scientific. ALG has received support for attending scientific meetings from Medtronic, Boston Scientific and Abbott.

## Supporting information


**Data S1.** Supporting Information.
**Figure S1.** The ROI‐tSSS method is extended to cuboidal rather than purely spherical ROIs. Here we consider the integration bounds of a cuboidal ROI of x, y and z dimensions 10 cm, 4 cm and 2 cm. The shape can be split into two pairs of identical prisms, which are serially added moving from A through to D, giving rise to the total volume. Regularly spaced coordinates selected along the integration bounds $\left(r,\varphi ,\theta \right)$ in the spherical coordinate system are transformed into cartesian coordinates for plotting. The green point indicates the origin (0,0,0).

## Data Availability

Phantom recording data used in this paper are available at the following repository: https://figshare.com/articles/dataset/phantom090715_BrainampDBS_20150709_01_ds_zip/4042911. Code for implementing the ROI‐tSSS algorithm are shared on GitHub repository https://github.com/AshOswal/ROI-tSSS.
