# Supplementary figures and images for: Spatiotemporal signal space separation for regions of interest: Application for extracting neuromagnetic responses evoked by deep brain stimulation
Source: Hum Brain Mapp. 2024 Jan 30;45(2):e26602. doi: 10.1002/hbm.26602 (PMC10826894; doi:10.1002/hbm.26602)

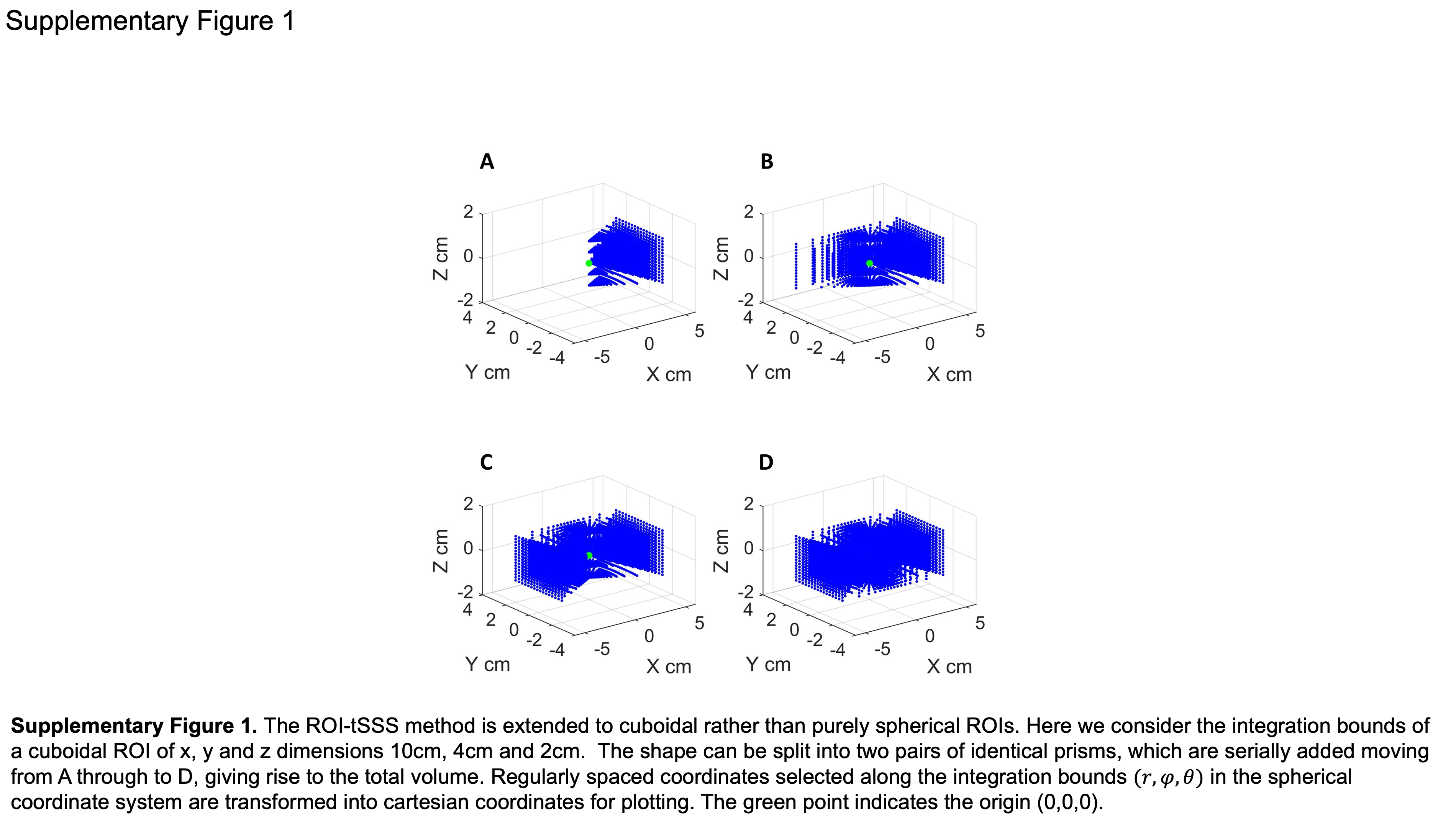

Supplement: Supplementary file 1 — Data S1. Supporting Information. Figure S1. The ROI‐tSSS method is extended to cuboidal rather than purely spherical ROIs. Here we consider the integration bounds of a cuboidal ROI of x, y and z dimensions 10 cm, 4 cm and 2 cm. The shape can be split into two pairs of identical prisms, which are serially added moving from A through to D, giving rise to the total volume. Regularly spaced coordinates selected along the integration bounds rφθ in the spherical coordinate system are transformed into cartesian coordinates for plotting. The green point indicates the origin (0,0,0). [file HBM-45-e26602-s001.zip › hbm26602-sup-0002-FigureS1.tiff]
